# Draft Genome Sequence of the Brazilian *Cyanobium* sp. Strain CACIAM 14

**DOI:** 10.1128/genomeA.00669-14

**Published:** 2014-07-10

**Authors:** Alex Ranieri Jerônimo Lima, Andrei Santos Siqueira, Bruno Garcia Simões dos Santos, Fábio Daniel Florêncio da Silva, Clayton Pereira Lima, Jedson Ferreira Cardoso, João Lídio da Silva Gonçalves Vianez Júnior, Leonardo Teixeira Dall'Agnol, John Anthony McCulloch, Márcio Roberto Teixeira Nunes, Evonnildo Costa Gonçalves

**Affiliations:** a Laboratório de Tecnologia Biomolecular, Instituto de Ciências Biológicas (ICB), Universidade Federal do Pará (UFPA), Belém, Pará, Brazil; b Centro de Inovações Tecnológicas (CIT), Instituto Evandro Chagas (IEC), Ministério da Saúde, Ananindeua, Brazil

## Abstract

Given the scarcity of data pertaining to whole-genome sequences of cyanobacterial strains isolated in Brazil, we hereby present the draft genome sequence of the *Cyanobium* sp. strain CACIAM 14, isolated in southeastern Amazonia.

## GENOME ANNOUNCEMENT

Given their ability to synthesize a wide variety of biologically active products (1–3), the cyanobacteria have played a relevant role in modern biotechnology. Their full biotechnological potential (4, 5) might be more easily exploited with knowledge of genetic content. We have reconstructed the genome of a cyanobacterial species by means of a bioinformatics pipeline applied to reads obtained from a non-axenic culture of a *Cyanobium* sp. Data are scarce pertaining to genomes of cyanobacteria isolated in Brazil, with currently only a few strains sequenced (6, 7).

We have hereby applied a next-generation sequencing pipeline according to Albertsen et al. (8) in order to obtain the genomic data pertaining to the *Cyanobium* sp. strain CACIAM 14, a unicellular cyanobacterium which was isolated from a water sample collected in December 2010 in the Tucuruí Hydroelectric Dam (3°49′55″S, 49°38′50″W) in the State of Pará, Brazil.

Two genomic DNA samples cultured 6 months apart were obtained from a non-axenic cyanobacterial biomass. The two nonpaired libraries were sequenced using the GS FLX 454 platform (Roche Life Science), yielding 660,228 (~255 Gb) and 815,325 (~357 Gb) reads for the first and second runs, respectively.

The datasets were assembled separately with Newbler 2.6 (minimum read size, 45 bp; minimum overlap, 40 bp; minimum overlap identity, 90%). These assemblies generated 3,654 and 3,256 contigs larger than 1 kb in length, with *N*_50_ values of 2,149 bp and 37,998 bp.

The contigs were identified and separated using a metagenomic assembling pipeline (8) for each putative organism, i.e., the isolated cyanobacterium and its associated heterotrophic bacterium (9, 10). The assembled contigs from the second run were used to determine the genome coverage.

Our analyses permitted the recovery of a draft genome which contains 71 contigs (total of ~3.2 Mb), ranging from 5,275 to 403,256 bp. The average coverage was 25×, with an assembly *N*_50_ of 61,937 bp and GC content of 68.56%.

The pipeline (8) used applied 107 hidden Markov models for essential genes present in a single copy in 95% of all bacteria. The genome draft sequence we present contains 108 of these genes, including duplications of the TIGR00436 and TIGR02350 genes. There is usually duplication of TIGR00436 only.

Structural annotation was carried out with the Prokaryotic Genome Annotation Pipeline (PGAP) tool, available on the NCBI website (11), resulting in 2,935 annotated coding sequences (CDSs) and 40 tRNA genes. The rRNA clusters were predicted by the RNAmmer tool (12). 16S rRNA was, however, found to be missing from this version of the draft sequence. Taxonomical identification was thus carried out based on the polymorphisms of the alpha and beta subunits of phycocyanin (according to Dall’Agnol et al. [13]), which presented 93% and 88% nucleotide identity, respectively, to those of *Cyanobium gracile* PCC 6307.

A preliminary analysis of the draft genome sequence using the antiSMASH tool (14) revealed the presence of 3 terpene clusters and 4 bacteriocin synthesis clusters.

### Nucleotide sequence accession numbers.

This whole-genome shotgun project has been deposited at DDBJ/EMBL/GenBank under the accession number JMRP00000000. The version described in this paper is version JMRP01000000.

## References

[1] Beck C Knoop H Axmann IM Steuer R 2012 The diversity of cyanobacterial metabolism: genome analysis of multiple phototrophic microorganisms. BMC Genomics 13:56. 10.1186/1471-2164-13-56 22300633 PMC3369817

[2] Hess WR 2011 Cyanobacterial genomics for ecology and biotechnology. Curr. Opin. Microbiol. 14:608–614. 10.1016/j.mib.2011.07.024 21840247

[3] Abed RM Dobretsov S Sudesh K 2009 Applications of cyanobacteria in biotechnology. J. Appl. Microbiol. 106:1–12. 10.1111/j.1365-2672.2008.03918.x 19191979

[4] Englund E Pattanaik B Ubhayasekera SJK Stensjö K Bergquist J Lindberg P 2014 Production of squalene in *Synechocystis* sp. PCC 6803. PLoS One 9:e90270. 10.1371/journal.pone.0090270 24625633 PMC3953072

[5] Wang H Fewer DP Sivonen K 2011 Genome mining demonstrates the widespread occurrence of gene clusters encoding bacteriocins in cyanobacteria. PLoS One 6:e22384. 10.1371/journal.pone.0022384 21799841 PMC3140520

[6] Fiore MF Alvarenga DO Varani AM Hoff-Risseti C Crespim E Ramos RTJ Silva A Schaker PDC Heck K Rigonato J Schneider PC 2013 Draft genome sequence of the Brazilian toxic bloom-forming cyanobacterium *Microcystis aeruginosa* strain SPC777. Genome Announc. 1(4):e00547-13. 10.1128/genomeA.00547-13 23908289 PMC3731843

[7] Stucken K John U Cembella A Murillo AA Soto-Liebe K Fuentes-Valdés JJ Friedel M Plominsky AM Vásquez M Glöckner G 2010 The smallest known genomes of multicellular and toxic cyanobacteria: comparison, minimal gene sets for linked traits and the evolutionary implications. PLoS One 5:e9235. 10.1371/journal.pone.0009235 20169071 PMC2821919

[8] Albertsen M Hugenholtz P Skarshewski A Nielsen KL Tyson GW Nielsen PH 2013 Genome sequences of rare, uncultured bacteria obtained by differential coverage binning of multiple metagenomes. Nat. Biotechnol. 31:533–538. 10.1038/nbt.2579 23707974

[9] Lima ARJ Siqueira AS dos Santos BGS da Silva FDF Lima CP Cardoso JF Vianez-Júnior JLSG Nunes MRT Gonçalves EC 2014 Draft genome sequence of *Blastomonas* sp. strain CACIA 14H2, a heterotrophic bacterium associated with cyanobacteria. Genome Announc. 2(1):e01200-13. 10.1128/genomeA.01200-13 24435876 PMC3894290

[10] Lima ARJ Siqueira AS dos Santos BGS da Silva FDF Inada DT Lima CP Cardoso JF Vianez-Júnior JLSG Nunes MRT Gonçalves EC 2014 Draft genome sequence of *Rhodobacter* sp. strain CACIA 14H1, a heterotrophic bacterium obtained from a nonaxenic culture of a *Cyanobium* species. Genome Announc. 2(1):e01116-13. 10.1128/genomeA.01116-13 24435858 PMC3894272

[11] Angiuoli SV Gussman A Klimke W Cochrane G Field D Garrity G Kodira CD Kyrpides N Madupu R Markowitz V Tatusova T Thomson N White O 2008 Toward an online repository of standard operating procedures (SOPs) for (meta)genomic annotation. Omics 12:137–141. 10.1089/omi.2008.0017 18416670 PMC3196215

[12] Lagesen K Hallin P Rødland EA Staerfeldt HH Rognes T Ussery DW 2007 RNAmmer: consistent and rapid annotation of ribosomal RNA genes. Nucleic Acids Res. 35:3100–3108. 10.1093/nar/gkm160 17452365 PMC1888812

[13] Dall'Agnol LT Ghilardi-Junior R Mcculloch Ja Schneider H Schneider MPC Silva A 2012 Phylogenetic and gene trees of *Synechococcus*: choice of the right marker to evaluate the population diversity in the Tucurui Hydroelectric Power Station Reservoir in Brazilian Amazonia. J. Plankton Res. 34:245–257. 10.1093/plankt/fbr109

[14] Blin K Medema MH Kazempour D Fischbach MA Breitling R Takano E Weber T 2013 antiSMASH 2.0—a versatile platform for genome mining of secondary metabolite producers. Nucleic Acids Res. 41:W204–W212. 10.1093/nar/gkt449 23737449 PMC3692088

